# Study of Optimal Perimetric Testing in Children (OPTIC): Feasibility, Reliability and Repeatability of Perimetry in Children

**DOI:** 10.1371/journal.pone.0130895

**Published:** 2015-06-19

**Authors:** Dipesh E. Patel, Phillippa M. Cumberland, Bronwen C. Walters, Isabelle Russell-Eggitt, Jugnoo S. Rahi, OPTIC study group

**Affiliations:** 1 Life Course Epidemiology and Biostatistics Section, UCL Institute of Child Health, London, United Kingdom; 2 Ulverscroft Vision Research Group, London, United Kingdom; 3 Moorfields Eye Hospital NHS Foundation Trust, London, United Kingdom; 4 Great Ormond Street Hospital for Children NHS Foundation Trust, London, United Kingdom; 5 UCL Institute of Ophthalmology, London, United Kingdom; University of Waterloo, CANADA

## Abstract

**Purpose:**

To investigate feasibility, reliability and repeatability of perimetry in children.

**Methods:**

A prospective, observational study recruiting 154 children aged 5–15 years, without an ophthalmic condition that affects the visual field (controls), identified consecutively between May 2012 and November 2013 from hospital eye clinics. Perimetry was undertaken in a single sitting, with standardised protocols, in a randomised order using the Humphrey static (SITA 24–2 FAST), Goldmann and Octopus kinetic perimeters. Data collected included test duration, subjective experience and test quality (incorporating examiner ratings on comprehension of instructions, fatigue, response to visual and auditory stimuli, concentration and co-operation) to assess feasibility and reliability. Testing was repeated within 6 months to assess repeatability.

**Results:**

Overall feasibility was very high (Goldmann=96.1%, Octopus=89% and Humphrey=100% completed the tests). Examiner rated reliability was ‘good’ in 125 (81.2%) children for Goldmann, 100 (64.9%) for Octopus and 98 (63.6%) for Humphrey perimetry. Goldmann perimetry was the most reliable method in children under 9 years of age. Reliability improved with increasing age (multinomial logistic regression (Goldmann, Octopus and Humphrey), *p*<0.001). No significant differences were found for any of the three test strategies when examining initial and follow-up data outputs (Bland-Altman plots, *n*=43), suggesting good test repeatability, although the sample size may preclude detection of a small learning effect.

**Conclusions:**

Feasibility and reliability of formal perimetry in children improves with age. By the age of 9 years, all the strategies used here were highly feasible and reliable. Clinical assessment of the visual field is achievable in children as young as 5 years, and should be considered where visual field loss is suspected. Since Goldmann perimetry is the most effective strategy in children aged 5–8 years and this perimeter is no longer available, further research is required on a suitable alternative for this age group.

## Introduction

Visual field (VF) testing is a key parameter in assessing and monitoring visual function in patients with ophthalmic and neurological diseases[1]. It is estimated that over 3500 children under 16 years of age undergo formal perimetry in the UK per year[2], without any consensus on approaches in children. There is also a paucity of robust data on the correct interpretation of these tests, in particular on reliability, to inform understanding of the usefulness of perimetry in monitoring children.

To date, a number of small studies have investigated perimetry in children without ophthalmic conditions[3–10], generally using methods and algorithms not commonly available/utilised in routine clinical practice. Variation in findings relating to test feasibility and reliability reflects the diversity of testing strategies.

In UK hospitals, the Humphrey SITA algorithms and Goldmann perimetry (no longer commercially available) are the two most common perimetric approaches in children with suspected/confirmed VF loss[2]. Prior studies have tested feasibility of Goldmann perimetry using single isopters with large stimuli (i.e. V4e or III4e) along limited test meridians, limiting their ability to inform clinical practice[9–12]. Semi-automated kinetic perimetry (Octopus 900) is reported to be feasible in children[13]. However there is no evidence regarding its comparative feasibility and reliability, which is necessary to understand whether the Octopus can reliably replace the Goldmann as the perimeter of choice in children.

The SITA algorithms are some of the shortest threshold tests available[11] and children are able to perform shorter static algorithms more reliably than longer tests[14, 15], with the SITA Fast and Standard algorithms having equivalent precision for detecting VF progression[16]. Assessment of reliability in static perimetry currently relies on the use of automated indices (false positives, fixation losses, and false negatives) despite evidence reported in adults that reproducibility (the gold-standard measure of reliability) is not associated with any of these measures[17]. Thus assessment of reliability and subsequent interpretation of results in children using static perimetry is currently unknown.

There is therefore a limited evidence base on which clinicians can draw to decide which perimetric technique to use, how to assess test reliability and interpret the findings accounting for subject age. As part of a larger programme of research investigating the role of paediatric perimetry, we report here an investigation of feasibility, reliability, and repeatability of common perimetric tests in children. Specifically, we compared Octopus automated kinetic perimetry with the current ‘gold-standard’ Goldmann kinetic perimetry.

## Methods

Participants (described in Table 1) were recruited consecutively from patients and their siblings attending Moorfields Eye Hospital, London, reflecting the broader patient population of children who might require visual field testing as part of their clinical care. Parents or legal guardians gave written consent for participation and children gave verbal assent. Ophthalmic diagnosis, visual acuity and refractive state were extracted from clinical case notes. Children without medical records (siblings of patients) underwent a full Orthoptic examination, including focimetry where appropriate.

**Table 1:** Inclusion/exclusion criteria for participants.

***Inclusion Criteria***
Children aged 5 to 15 years
No history of ophthalmological disease that could cause a visual field defect, but including children with refractive error, unilateral amblyopia and strabismus, where the fellow (normal) eye was to be tested. No prior experience of perimetry.
Visual acuity of 0.2 LogMAR or better (20/32 Snellen equivalent) in the tested eye
***Exclusion Criteria***
Children with any impairments, such as severe learning disability, which would make co-operation with formal perimetry challenging
Children not accompanied by a parent or legal guardian

Assessments were performed using an Octopus 900 (Haag-Streit AG, Switzerland), a Humphrey Visual Field Analyzer 750i (Carl Zeiss Meditec VG mbH, Germany) and a Goldmann perimeter (Haag-Streit, Bern, Switzerland). All tests were carried out by an experienced Orthoptist (DEP) in the same visual field testing room fitted with a blackout blind and using regularly calibrated perimeters. Assessments were undertaken in a randomised order (assigned by a random number generator, Microsoft Excel 2010), with short rest periods between tests. In children with unilateral amblyopia, the non-amblyopic eye was tested. For those with good acuity in each eye, and no strabismus/treated amblyopia, one eye was randomly selected and tested.

At the start of the session, the participant sat on a height adjustable chair and he/she was shown the relevant perimeter prior to testing and was given an explanation of the test procedure. This involved instructions to fix centrally and press their buzzer every time a light was perceived (either a flash or moving light dependent on the perimeter). He/she was also given an opportunity to test the buzzer. All instructions were delivered in age appropriate language. The child then had the non-tested eye occluded with a soft eye pad, was set up at the perimeter, and seat and chin rest adjustments were made until the position was correct and he/she felt comfortable. Additional padding on the chinrest to reach the correct height was given to any participant requiring it. Significant refractive errors were corrected using large aperture lenses for the I2e stimulus and static perimetry only using criterion modified from Henson[18]: ≥ +3.00 dioptre spheres (DS), ≥ -1.00DS, > ±1.00 dioptre cylinders (DC). The time taken to prepare the participant was recorded and a note was made of any modifications necessary to perform the assessment. Encouragement and repetition of instructions were given throughout the tests. Participants were offered a rest break during the test if they appeared to be getting tired/losing concentration and if taken, this was recorded by the examiner.

### Kinetic visual field assessments

Both the Goldmann and Octopus kinetic perimetry assessments were performed using the same testing protocol, adapted from Werner[19]. Assessments started with three practice presentations with the first test isopter allowing familiarisation with the test procedure. These were not used to form the test isopter. Targets were then presented along 12 cardinal meridian (every 30° in a pre-defined order), centripetally from a non-seeing area. Test points were started at manually plotted locations on the Octopus, with an automated speed of 5°/sec[13].

After plotting the 12 cardinal points, additional points were tested, in those children able to tolerate more extensive testing, in a non-randomised order along meridians 15° adjacent to the cardinal points starting with temporal field locations. This effectively ‘filled-in’ areas with larger distances between test points first, and allowed for more accurate plotting of visual field shape, up to a maximum of 24 points per isopter. Two isopters were plotted, to replicate common clinical practice and avoid overburdening participants, the choice being randomised between III4e, I4e and I2e.

Participants were asked to “sit back and relax” between isopters, allowing for a very short rest (generally less than 20 seconds). During this period they were shown the next test stimulus and then re-positioned to continue with the assessment.

The test procedure started with plotting an outer isopter, followed by inner isopter and then finally the plotting of the blind spot, with the I2e stimulus (stimulus speed of 2°/sec). This allowed children to get accustomed to testing using an easier stimulus, and allowed children to relate to an increased difficulty between isopters as “moving on to the next level.”

Test points were re-plotted if the examiner felt the initial response was unreliable, to allow for temporary lapses in concentration and co-operation, without masking persistently limited co-operation.

### Humphrey static perimetry assessment

Participants were assessed with the SITA 24–2 FAST algorithm. Gaze-tracking and blind spot monitoring were attempted using the Heijl-Krakau method.

Participants were specifically warned the lights could be “really bright or quite hard to see” during this test and were told when they were halfway through the algorithm.

### Examiner Based Assessment of Reliability (EBAR)

Participants were rated on each perimetric assessment using an Examiner Based Assessment of Reliability (EBAR) scoring system developed for this study. The EBAR score is a qualitative, categorical system with outcomes of ‘good’, ‘fair’ or ‘poor’ quality of perimetric test. It is independent of visual field outcome. The EBAR rating was designed and implemented to guide the evaluation of reliability in paediatric perimetry. Participants were assigned a score using the criteria in Table 2.

**Table 2:** Examiner Based Assessment of Reliability (EBAR) scoring system.

**‘Good’ rating:** Compliance with testing is good. The participant is able to maintain good central fixation and respond promptly. They may have some fixation losses at times, but are able to understand and comply well with test instructions. General behaviour allows a comprehensive assessment and overall, visual field outcome is expected to represent true visual field size/sensitivity.
**‘Fair’ rating:** Compliance with testing is mostly good. The participant may have moderate fixation losses with some variability in responses. They are able to understand test instructions and their general behaviour allows for moderate co-operation. They may show evidence of fatigue that affects performance and respond to the noise of stimulus presentation at times. Overall, visual field outcome is expected to be able to detect gross defects, but may over/under-estimate true visual field size/sensitivity.
**‘Poor’ rating:** Compliance with testing is poor. The participant demonstrates very high fixation losses or searching for stimuli. They may be unable to ignore the sound of stimulus presentation and therefore produce high false positive responses. They may also demonstrate highly variable responses, with a possible lack of understanding of test instructions. Overall, test performance is not expected to represent true visual field size/sensitivity and results will be unable to rule-in or rule-out visual field defects.

The time taken for each perimetric assessment, any modifications required, use of an additional chinrest or the need for rest breaks during the assessment were noted. Subjects who found it difficult to keep their chin resting at the perimeter were supported to keep their heads in position, but it did not impact on EBAR rating unless associated with other factors (e.g. poor concentration).

### Assessment of subjective experience of perimetry

Participants rated how difficult they found each assessment, using a 5-point Likert scale ranging from ‘Very Hard’ to ‘Very Easy’. Any other comments were recorded verbatim.

### Follow-up examination

All participants were invited to return for a repeat examination within 6 months of the original testing to investigate repeatability. All repeat procedures were carried out in the same manner and order as the initial test.

### Statistical methods

Raw data were extracted from the Humphrey perimeter using R code developed at City University (personal communication: Richard Russell) (The R Project for Statistical Computing (R v3.0.3, www.r-project.org)). Raw Goldmann and Octopus data were compiled using the R package‘kineticF’[20] (R v3.1.2, www.r-project.org/).

Paper records completed by the examiner were entered into the database software REDCap[21] (Research Electronic Data Capture) hosted securely at UCL Institute of Child Health. All other analyses were performed in STATA (StataCorp. 2011. *Stata Statistical Software*: *Release 12*. College Station, TX: StataCorp LP). All left eye data were mirrored along the y-axis to represent right eye data for analysis. Chi-squared tests, linear and multinomial logistic regression analyses were undertaken.

The study was approved by the National Health Service Research Ethics Committee for London—Bloomsbury and followed the tenets of the Declaration of Helsinki.

## Results

One hundred and fifty-four participants (Table 3) were tested between May 2012 and November 2013 from 348 eligible participants (44.3%). Of these, 43 (27.9%) returned for a follow-up visit.

**Table 3:** Participant demographics and test feasibility for all perimeters (n = 154).

Age group (years)	Sex		Number completing assessments (%)			Mean test duration* (min) (SD)		
	*Male*	*Female*	*Goldmann*	*Octopus*	*Humphrey*	*Goldmann*	*Octopus*	*Humphrey*
**5–6**	22	18	36 (90)	32 (80)	40 (100)	9.2 (1.9)	9.1 (1.44)	7 (1.3)
**7–8**	23	30	51 (96.2)	48 (90.6)	53 (100)	9.4 (1.8)	9.1 (1.8)	6.2 (1.0)
**9–11**	13	22	35 (100)	32 (91.4)	35 (100)	9.3 (1.3)	8.5 (1.0)	5 (1.0)
**12–15**	15	11	26 (100)	25 (96.2)	26 (100)	8.6 (1.1)	8 (0.9)	4.6 (0.7)

*Test duration values include preparation and assessment tasks

Most (*n* = 93, 60.4%) were aged 5 to 8 years and 118/154 (76.6%) were White, with 9.7% Indian, 8.4% Black and 5.2% of mixed ethnicity. Those declining to participate were of similar ethnicity and age to those participating.

Median refractive error was 0.00D (spherical equivalent) IQR = 0D to +2.50D, range = -10.00 to +6.75D. 56/154 (36.4%) participants had strabismus, and 35/154 (22.7%) had unilateral amblyopia. Unless otherwise stated, reliability refers to results from EBAR (subjective examiner) scoring.

### Feasibility of perimetry in children

No child had to stop testing completely due to fatigue or an unwillingness to continue with the assessment. All 3 perimetric tests were highly feasible for all ages (Table 3).

Increasing test quality (from ‘poor’ to ‘good’ quality) reduced test duration for all three perimeters (Goldmann (*p*<0.001), Octopus *(p* = 0.035), and Humphrey (*p*<0.001)).

For Octopus and Humphrey perimetry, 13/154 (8.4%) children, all under 8 years of age, required the use of additional chinrest support for correct positioning at the perimeter. Only 1/154 (0.7%) child required modifications to be successfully aligned for Goldmann perimetry (sat up on knees to reach the required height). Only 8/154 (5.2%) of all children showed visible signs of fatigue for Humphrey perimetry compared to 13/154 (8.4%) performing Goldmann and 19/154 (12.3%) performing Octopus assessments. No child above 9 years was affected by fatigue for Goldmann and Humphrey perimetry. Only 1/154 (0.7%) child, aged 7 years, required a break during Goldmann perimetry, with 4/154 (2.6%) and 9/154 (5.8%) requiring breaks for Octopus and Humphrey perimetry respectively.

**Fig 1:**
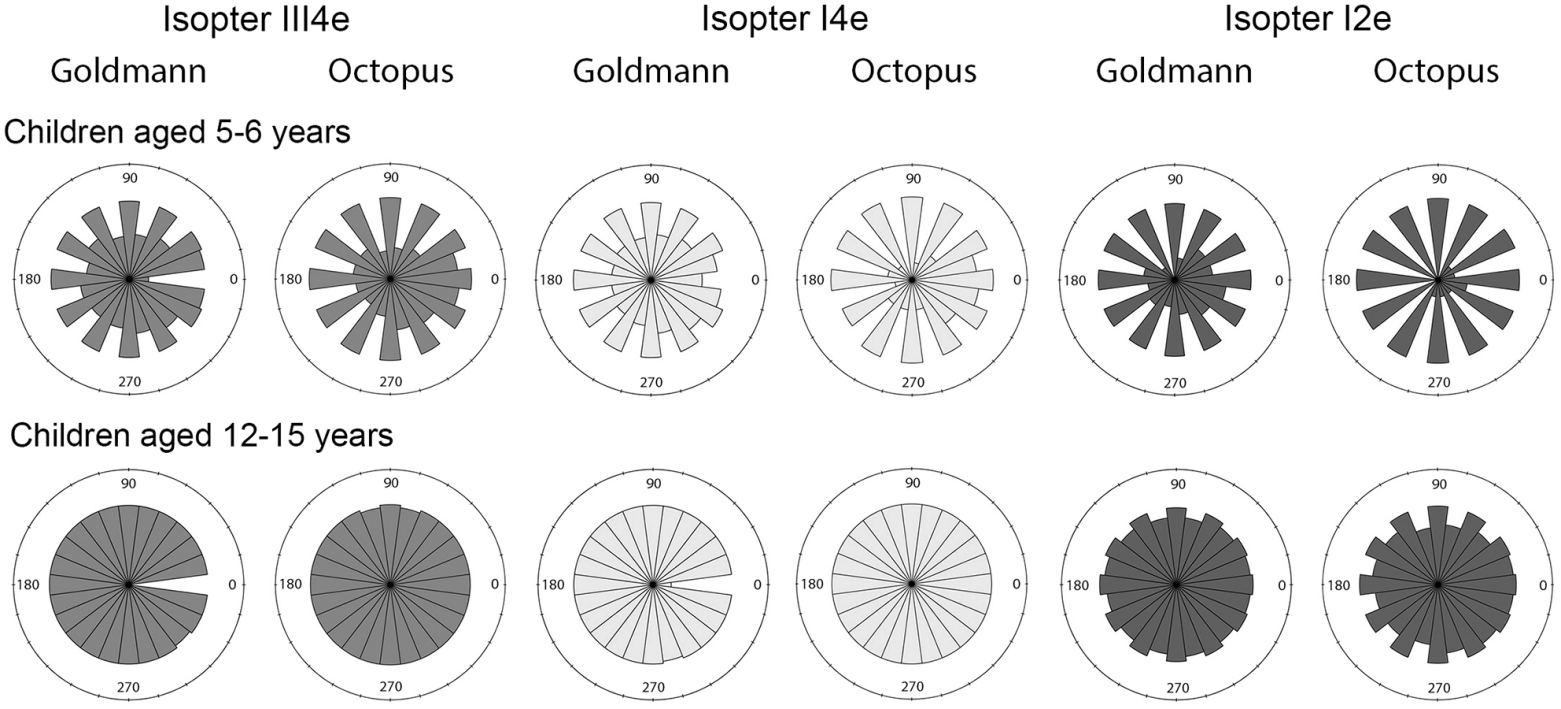
Rose diagrams of the frequency of points plotted along individual meridians for Goldmann and Octopus perimetry for children aged 5–6 years compared to 12–15 years. A larger area indicates a meridian with a larger number of plotted points. *The empty sectors at 0° for Goldmann perimetry isopters III4e and I4e correspond to the ‘void’ area in the perimeter bowl.

For all isopters on both kinetic perimeters, there was a statistically significant increase in the number of points that could be plotted per isopter with age (*p*<0.0001) (Fig 1).

### Reliability of perimetry in children

**Fig 2:**
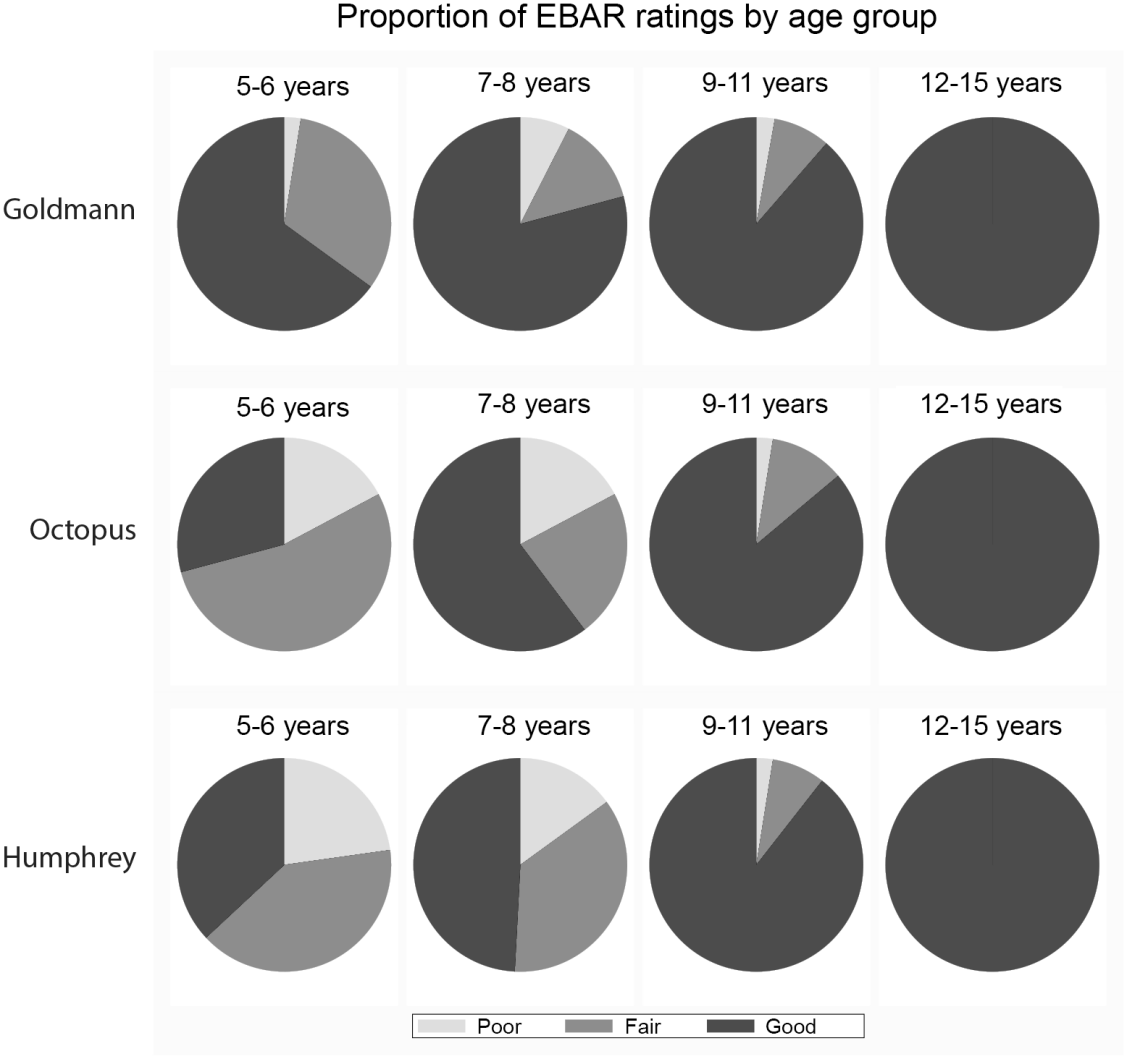
Proportion of EBAR (test quality) ratings per perimeter, by age groups.

Fig 2 demonstrates the change in the proportion of ‘good’ EBAR ratings (test quality) with age for each perimetric assessment. Only Goldmann perimetry had >50% of tests rated as ‘good’ for children aged 5–6 years but test reliability improved with age for all perimeters (*p*<0.0001). By ages 7–8 years there is a shift from large proportions of ‘fair’ tests to ‘good’ quality tests. In children over 9 years of age, no significant difference was found between Goldmann and Humphrey assessments (χ^2^, *p* = 0.123).

Traditional reliability indices (RI) (fixation losses ≥ 25% or false positives ≥15%, as recorded by the Humphrey perimeter) indicated a large number of assessments (125/154 (81.2%)) would be classified as unreliable. Traditional RI’s disagreed with EBAR in 74/154 cases (48.1%) (Table 4) i.e. 72 (good rating & unreliable RI) + 2 (poor rating & reliable RI). Splitting the two variables that compose the reliability index shows that fixation losses alone show a similar pattern of unreliability, and poor agreement with EBAR (test for trend; *p* = 0.196). However, only 42/154 (27%) assessments would be classified as unreliable using false positives alone and there is better agreement with EBAR, (test for trend; *p*<0.001), with only 17/154 (11%) assessments showing disagreement.

**Table 4:** Comparison of Examiner Based Assessment of Reliability (EBAR) with automated reliability indices for Humphrey perimetry.

EBAR Rating	False Positives		Fixation Losses		Traditional Reliability Indices*	
	*<15%*	*≥15%*	*<25%*	*≥25%*	*Reliable*	*Unreliable*
**Good**	87	11	30	68	26	72
**Fair**	19	19	1	37	1	37
**Poor**	6	12	6	12	2	16
**Total**	112	42 (27%)	37	117 (76%)	29	125 (81%)

*Traditional reliability indices are defined here as fixation losses ≥ 25% or false positives ≥15%

Examiner report showed in 16/154 (10.4%) (Goldmann) and 45/154 (29.2%) (Octopus) children it was not possible to reliably plot the blind spot. Of these 10/16 (62.5%) and 23/45 (51.1%) had tests rated ‘fair’ or ‘poor’ quality for Goldmann and Octopus respectively.

Participants reported testing with the Humphrey perimeter to be the most difficult (24.2% rated as difficult) and with the Goldmann to be the easiest (63.3% rated as easy). No relationship was found between subjective experience of test difficulty and examiner-rated test quality (Goldmann (*p* = 0.305), Octopus (*p* = 0.146) and Humphrey (*p* = 0.166)).

Notably, 39/154 (25.3%) of all children, mostly those aged ≤8 years (33/39), commented on, or responded to, the audible noise of stimulus presentation for Octopus perimetry. Of these, 17 (11% of the total sample) were reported by the examiner to have impacted on test quality. Verbatim responses from 33/154 (21.4%) participants using Humphrey perimetry were categorised and showed factors such as the rapid rate of stimulus presentation, the effect of testing to threshold (lots of stimuli that were difficult to see), and the varying locations of stimuli to be responsible for perceived difficulty.

### Repeatability of perimetry in children

43/154 (27.9%) of children returned for a repeat assessment within 6 months of the original test with a mean follow-up time of 108 days (SD = 42). The follow-up sample had a similar age distribution to the initial sample (children aged 5–6 years (*n* = 16), 7–8 years (*n* = 13), 9–11 years (*n* = 7) and 12–15 years (*n* = 7)).

Bland-Altman plots of Goldmann and Octopus isopter areas for children with ‘good’ quality tests at both visits were performed and there were no statistically significantly differences found for any isopter, indicating good test repeatability on both perimeters (S1 Fig).

No significant difference in Humphrey perimetry mean deviation (MD) values were found between the two visits (Bland-Altman, Mean difference = -0.24dB (95%CI: [-3.6, 0.7]).

## Discussion

We report that good quality perimetry is feasible in children as young as 5 years, although the prospects of achieving a reliable test improve with increasing age. Goldmann perimetry is the most reliable form of testing up to 9 years of age, but there appear to be no differences in reliability between test strategies above this age. Reliable tests are reproducible on repeat testing. Older children are able to plot more detailed kinetic assessments, allowing for better delineation of isopter shape.

Currently, there are no standardised methods for scoring test reliability in kinetic perimetry—for adults or children. The development and use of a new qualitative score here (EBAR) allows for assessment of reliability in kinetic perimetry, as well as providing information complementary to automated indices from static perimetry. EBAR can also be compared to participants’ perception of test difficulty in children.

The lack of relationship between participants’ perceived test difficulty and test reliability underlines the importance of encouraging children through tasks they perceive as difficult. Static perimetry (SITA 24–2 FAST) was the shortest test used here, yet had the poorest reliability in young children and participant responses identified the intensity of the task as a potential cause of this.

Fatigue is known to impact on test reliability and outcomes[22]. For static perimetry (with threshold algorithms) this affects the accuracy of the entire test, whereas points plotted by kinetic perimetry before the onset of fatigue will still provide useful data. Thus, in a child who tires quickly, or struggles with intensive testing, plotting a baseline kinetic field can give valuable information on visual field sensitivity that cannot be achieved by static techniques. Our data suggest it is possible to find a balance between performing the test quickly (minimising fatigue) and ensuring children do not feel overwhelmed by the task.

Our protocols are suitable for use in a clinical setting, combining short familiarisation tasks and commonly available tests. By careful positioning[23], familiarising, and engaging the participant with the task[24], we were able to maximise the potential for a reliable result even in children as young as 5 years. Assessments were performed by a single, experienced examiner, and test feasibility and duration reflect this. Clinically, it is anticipated that children requiring visual field tests would be examined by clinicians experienced in performing perimetry in children. Our data also addressed gaps in the literature relating to the limits for number of points plotted per isopter (in kinetic perimetry) [11].

A small subset of participants returned for follow-up review, precluding our ability to assess repeatability in depth. However, we found ‘good’ results to be consistent (reproducible), and thus useful for indicating a change in visual field result occurring from a true change in sensitivity. It is expected that test reproducibility would differ if test quality varied between tests, and as such, these analyses are not presented. As with any study assessing subjective responses in children, our study was designed to capture as much relevant data in a single sitting without reducing the data quality from fatigue, or inducing sampling bias by use of protocols that subjects/families would be unwilling to undertake. Our data on children without visual field loss preclude our ability to comment specifically on test duration in the context of major field loss, which can be anticipated to require longer assessment [25]. Assessing three VF tests in one visit allowed for a greater breadth of comparisons but limited the testing to one eye. Nevertheless, we report here the largest systematic study of feasibility and reliability of perimetry in children assessing three key approaches.

We are unable to make direct comparisons with prior studies using Goldmann perimetry, as these have used 12 test points (4 meridian—repeated twice), with a mean test duration of 5.1 minutes[9] or have tested along 8 meridian with one isopter (mean duration = 11.06 min)[11]. This considerable variation reported in test duration with similar protocols highlights the potential influence of examiner experience in manual perimetry. We used a highly detailed protocol yet the mean test duration for Goldmann perimetry was 9.2 minutes (SD = 1.6) and 8.8 minutes (SD = 1.5) for Octopus perimetry, indicating that detail and quality can be achieved with a child-appropriate test duration.

A recent report on Octopus perimetry in children using a detailed test protocol has shown that, as children struggled to plot a blind spot, only 64% of those aged 10–12 years could plot reliable fields[13]. This contrasts with our study, in which children demonstrated better reliability. This may reflect the more nuanced assessment of reliability we used, compared to the pre-defined metrics of others, but could also reflect variation in assessment protocols between studies. There is very little evidence on repeatability of VF testing in children. The Behavioural Visual Field Test (BEFIE) is a screening test developed specifically for use in children. A large, longitudinal, retrospective study of this technique has shown that a proportion of children with VF defects show changes in sensitivity (including improvement) that are presumed to be learning/developmental effects[26]. Both our study and another[13] found no evidence of a significant ‘learning effect’. However, as it would be reasonable to expect some learning effects, especially with younger children and differentially in those with field defects as opposed to those with normal fields, this issue requires further investigation.

Visual field tests are a valuable diagnostic tool but are only one facet of a clinical examination, and care should be taken not to over/under-value individual test results[27]. Formal perimetry should be attempted in children with suspected VF loss and we suggest the reliability of these assessments can be documented using the EBAR scoring system we have developed, so that judgements can be made as to whether test results reflect true visual field sensitivity. The sole use of automated reliability measures for Humphrey static perimetry may lead to potentially useful results being disregarded and our data suggest false positive data can be combined with an EBAR score to better grade the reliability of individual test outputs.

For those children where formal perimetry is not possible, child-specific novel assessments have been suggested. These consist of supra-threshold tests using eye-tracking[28], or using modified perimeters[5]. Other techniques use game-based[29] or behavioural engagement[26]. These allow for a degree of quantification, but many of these require specialist equipment and are likely to only be performed in specialist centres.

Goldmann, Octopus and Humphrey perimetry are highly feasible in children, with Goldmann perimetry being the most reliable test in participants under 9 years of age. Above this age, all methods were highly reliable and normative, age-appropriate data exist for each perimetric technique[30]. Thus, the choice of perimetric technique should be informed by the clinical context of individual cases. The evidence base on repeatability remains incomplete and warrants further investigation to inform understanding of how to reliably rule-in/rule-out small but clinically significant changes in visual fields in children over time.

## Supporting Information

S1 Fig Bland-Altman plots of initial vs. follow-up visual field area. Bland-Altman plots of initial vs. follow-up visual field area for all isopters using Goldmann and Octopus perimetry.
(PDF)
