# Supplementary material for: Study of Optimal Perimetric Testing in Children (OPTIC): Feasibility, Reliability and Repeatability of Perimetry in Children
Source: PLoS One. 2015 Jun 19;10(6):e0130895. doi: 10.1371/journal.pone.0130895 (PMC4474916; doi:10.1371/journal.pone.0130895)
Supplement: S1 Fig — Bland-Altman plots of initial vs. follow-up visual field area for all isopters using Goldmann and Octopus perimetry. (PDF) [file pone.0130895.s001.pdf]

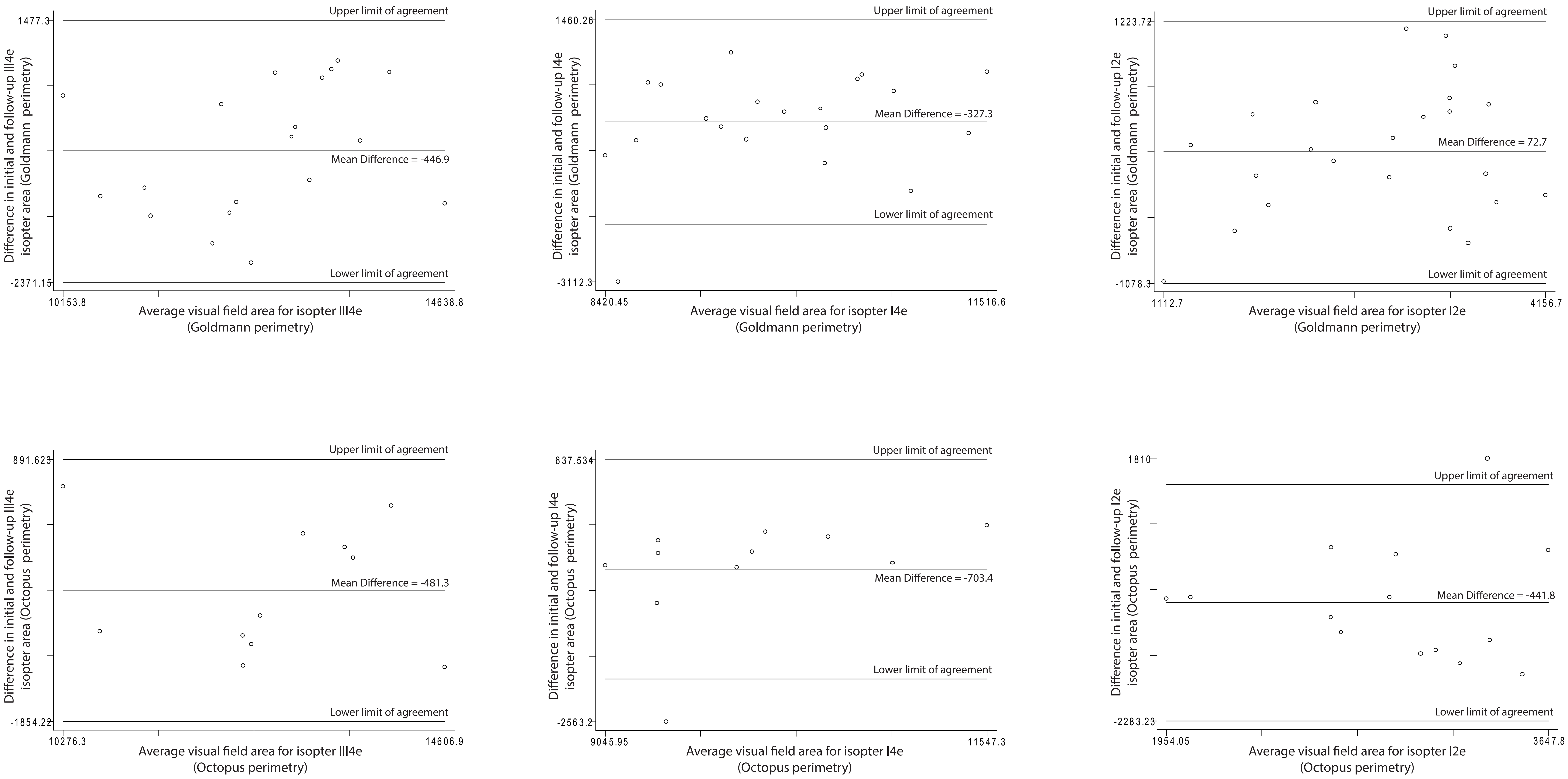

Supporting Information, Figure 1. Bland-Altman plots of initial vs. follow-up visual field area for all isopters using Goldmann and Octopus perimetry
